# Relationship of Susceptibility to Emotional Contagion with Automatic Emotion Processing and Emotional Competences

**DOI:** 10.3390/bs16050811

**Published:** 2026-05-19

**Authors:** Merle Welten, Anette Kersting, Thomas Suslow

**Affiliations:** Department of Psychosomatic Medicine and Psychotherapy, Medical Faculty, University of Leipzig, 04103 Leipzig, Germany; merle.welten@medizin.uni-leipzig.de (M.W.); anette.kersting@medizin.uni-leipzig.de (A.K.)

**Keywords:** affective priming, alexithymia, automatic evaluative shifts, emotion contagion, emotional intelligence, perceiving emotion, social emotion management, susceptibility to negative emotion contagion, susceptibility to positive emotion contagion, understanding emotion

## Abstract

Individuals differ in their susceptibility to emotional contagion, i.e., the automatic tendency to mirror and synchronize another person’s expressions and movements, resulting in shared emotional experiences. The objective of this research was to investigate how susceptibility to emotional contagion connects to automatic facial emotion processing and emotional competences. An affective priming task using happy, angry, neutral, and blank faces was administered to a sample of 104 women with a mean age of 24.72 years (SD = 3.63). They completed self-report measures assessing susceptibility to positive and negative emotional contagion, alexithymia, trait emotional intelligence, affectivity, and depression. Although prime valence-congruent evaluative shifts were found in our sample, there were no correlations of susceptibility to positive and negative emotional contagion with affective priming effects. Susceptibility to positive emotion contagion was negatively correlated with alexithymia and positively with emotional intelligence. However, susceptibility to positive emotion contagion predicted only emotional intelligence (but not alexithymia), when controlling for relevant affect variables. Our findings indicate that emotional contagion susceptibility could be less strongly linked to automatic emotion perception than previously suggested. Moreover, the trait-like tendency to resonate with other people’s positive emotions seems to be linked to enhanced capacities in perceiving, interpreting, and regulating emotions.

## 1. Introduction

Emotional contagion plays a crucial role in everyday life. From shared laughter to collective anxiety, individuals continuously resonate with their social environment. These emotional interactions are fundamental to social functioning, well-being and mental health (Frenzel et al., 2018; Kurtz & Algoe, 2015; Richer et al., 2024). The construct of emotional contagion was originally defined by Elaine Hatfield as “… the tendency to automatically mimic and synchronize facial expressions, vocalizations, postures, and movements with those of another person’s and, consequently, to converge emotionally” (Hatfield et al., 1993, p. 96). Put differently, emotional contagion refers to an automatic and unintentional “catching” of another individual’s emotions. One suspected mechanism underlying emotional contagion susceptibility might be facial mimicry: the spontaneous imitation of another person’s facial expression (Hatfield et al., 2014). Such motor resonance is assumed to generate afferent feedback, which in turn elicits a corresponding emotional experience in the observer (Coles et al., 2019; Strack et al., 1988). Importantly, individuals differ in the tendency to catch others’ emotions, i.e., their susceptibility to emotional contagion (Hatfield et al., 1993; Marx et al., 2024).

Individual differences in susceptibility to emotional contagion are commonly assessed using the Emotion Contagion Scale (ECS; Doherty, 1997), originally designed and validated as a unidimensional measure. Recent research, however, suggests that susceptibility to emotional contagion is better understood as a bi- or multidimensional construct, comprising separate sensitivities to positive and negative emotions (Clarkson et al., 2025; Lo Coco et al., 2014; Marx et al., 2024). Recently, Marx et al. (2024) developed a self-report measure that allows for a separate assessment of susceptibility to positive and negative emotional contagion. While emotional contagion is thought to involve automatic mimicry, little is known about how trait susceptibility to emotional contagion relates to the automatic perception of and responses to facial expressions.

An experimental paradigm suitable for investigating automatic processing of emotional facial expressions is affective priming. Affective priming refers to the phenomenon that individuals’ evaluations of neutral or ambiguous stimuli (targets) vary as a function of the affective valence of immediately preceding, only very briefly shown stimuli (primes) (Murphy & Zajonc, 1993). Numerous studies have demonstrated that targets are evaluated, for example, more positively when preceded by a positive facial expression than when preceded by a neutral or negative facial expression (Li et al., 2008; Paul et al., 2012; Sweeny et al., 2009). Affective priming effects can occur even when individuals are not aware of the prime stimuli (Murphy & Zajonc, 1993). Despite extensive research on affective priming, the mechanisms driving this phenomenon remain a subject of ongoing debate. Subcortical structures such as the amygdala and the nucleus accumbens may play an important role in automatic evaluative shifts (Suslow et al., 2013; Winkielman et al., 2007). Automatic facial mimicry is assumed to be involved in affective priming (Rotteveel et al., 2001), as activity of the musculus corrugator supercilii has been shown to mirror the valence of affective primes. Heightened corrugator activity is known to be indicative of negative affective states (Cacioppo et al., 1986; Topolinski & Strack, 2015). Against this background, affective priming effects may result from misattribution of feelings; the pleasant or unpleasant affect elicited by a prime could be transferred to the subsequently presented target (Rohr et al., 2015). However, it must be noted that affective priming effects might not be based primarily on sensorimotor feedback loops or associated affective reactions; rather, they may stem from cognitive appraisal processes (Wentura et al., 2017). The magnitude of affective priming effects appears to be decreased in alexithymic individuals (Rosenberg et al., 2020).

Alexithymia is a personality trait characterized by difficulties in processing and describing emotions (Luminet & Nielson, 2025). Alexithymic individuals typically exhibit diminished emotionalizing, i.e., reduced arousal and feelings in response to emotion-inducing stimuli or events (Pollatos et al., 2008; Vorst & Bermond, 2001), and also show decreased spontaneous facial reactions to other persons’ emotional facial expressions (Franz et al., 2021; Müller et al., 2019; Sonnby-Borgström, 2009). Consistent with these characteristics, negative correlations have been observed between alexithymia and susceptibility to emotional contagion (as assessed with the ECS) (Eddy & Hansen, 2021; Weisbuch et al., 2011). However, these studies correlated total scale scores, leaving unclear how susceptibility to positive and negative emotional contagion relates to individual facets of alexithymia.

While alexithymia represents deficits in emotional processing, susceptibility to emotional contagion may conversely promote the development of emotional abilities. Greater resonance and reactivity to emotions can enhance emotion recognition and regulation. Individuals highly susceptible to emotional contagion may therefore acquire more extensive emotion-related knowledge or rely more frequently on emotions to initiate or guide cognition. Conversely, individuals with high emotional intelligence may deliberately attenuate their susceptibility to negative emotional contagion in order to protect their own affective state. The abilities to perceive, use, understand, and regulate one’s own and others’ emotions in order to guide behavior and thinking have been conceptualized as emotional intelligence (Mayer & Salovey, 1997). To our knowledge, only one study examined the relationship between different forms of susceptibility to emotional contagion and emotional intelligence: Clarkson et al. (2025) observed in a sample of university students that susceptibility to positive emotional contagion was positively associated with emotional intelligence, while susceptibility to anxiety and fear contagion was negatively related to emotional intelligence (as assessed by the total score of the Wong and Law Emotional Intelligence Scale (Wong & Law, 2002)). It remains to be examined whether these differential relations can be confirmed by an independent study. Moreover, it should be explored which forms of susceptibility to emotional contagion are related to which facets of emotional intelligence.

The main objective of the present study was to investigate whether susceptibility to positive and negative emotional contagion is related to the automatic processing of facial emotions. For this purpose, we administered an affective priming task and examined evaluative shifts owing to masked emotional facial expressions. It can be argued that the affective priming task examines a form of susceptibility to being automatically influenced by facial emotions. Furthermore, we examined the relationships of susceptibility to positive and negative emotional contagion with the personality trait alexithymia and emotional intelligence, both of which focus on the capacity to recognize and process emotions and strongly influence emotional interpersonal functioning (Spitzer et al., 2005; Parker et al., 2021). The central hypotheses of our investigation were that susceptibility to positive emotional contagion is positively correlated with the affective priming effect based on happy faces and that susceptibility to negative emotional contagion is positively correlated with the affective priming effect based on angry faces. Moreover, we hypothesized that susceptibility to (positive and negative) emotional contagion is negatively correlated with alexithymia (cf. Eddy & Hansen, 2021; Weisbuch et al., 2011). Finally, based on Clarkson et al. (2025), we expected that susceptibility to positive emotional contagion is positively related to emotional intelligence and that susceptibility to negative emotional contagion is negatively related to emotional intelligence. Previous research reported sex differences in susceptibility to emotional contagion (Doherty, 1997; Doherty et al., 1995) and its relationships with emotional intelligence (Clarkson et al., 2025). For reasons of economic efficiency and to maintain a high level of statistical stringency, recruitment was limited to women. This allowed for the inclusion of a larger, more uniform participant pool, thereby increasing the reliability of the findings regarding emotional contagion susceptibility.

## 2. Materials and Methods

### 2.1. Participants

The sample consisted of 104 women. Their age ranged from 18 to 35 years (M = 24.72, SD = 3.63). The mean duration of participants’ school education was 12.12 years (SD = 0.58, range: 10–13). Most study participants (*n* = 66) were university students from different academic disciplines (63.5% of the sample). The other participants were attending high school (*n* = 1), in vocational training (*n* = 12), working (*n* = 16), unemployed (*n* = 6), on parental leave (*n* = 1) or in a period of waiting to take an entrance exam or start a new job (*n* = 2). Study participants were native German speakers and had normal (or corrected-to-normal) vision as assessed by a standard visual acuity chart. Pregnancy, age under 18 and over 35 years, self-reported past or actual presence of neurological, internistic or psychiatric diseases, and self-reported use of psychotropic substances were exclusion criteria. Doctoral students conducted initial screening calls to assess eligibility and were responsible for all subsequent data collection. They were trained in the administration of the screening interview and supervised by a senior clinical psychologist. Participants were recruited using a multimodal strategy, including an online study advertisement on the website of the Department of Psychosomatic Medicine, flyers posted on noticeboards in university libraries, and posts on social media platforms (public channels on Telegram, WhatsApp group chats, and Instagram). Study participants received compensation in the amount of €35. All procedures were approved by the Ethics Committee at the Medical Faculty of the University of Leipzig. All participants provided written consent after receiving information about the purpose and procedures of our study.

### 2.2. Questionnaires and Tests

The Positive and Negative Susceptibility to Emotional Contagion Scales (Positive SEC and Negative SEC; Marx et al., 2024) were developed to assess the trait-like tendency or proneness to catch other individuals’ emotions through the process of emotional contagion. The positive SEC subscale measures an individual’s susceptibility to positive emotional contagion, whereas the negative SEC subscale assesses susceptibility to negative emotional contagion. Both scales consist of four items and have demonstrated good reliability in terms of internal consistencies (Marx et al., 2024). The response scale of all items ranges from 1 to 5. There is evidence for a two-factor structure, with Positive SEC and Negative SEC as distinct yet correlated factors (Marx et al., 2024). In our sample, the mean Positive SEC item score was 4.57 (SD = 0.45) and the mean Negative SEC item score was 3.32 (SD = 0.76). In the study of Marx et al. (2024), internal consistencies (Cronbach’s alpha) were 0.76 and 0.82 for the negative SEC subscale and 0.83 and 0.83 for the positive SEC subscale. In our sample, internal consistency coefficients were somewhat lower: 0.75 for the negative SEC subscale and 0.67 for the positive SEC subscale.

The 20-Item Toronto Alexithymia Scale (TAS-20; German version: Bach et al., 1996) was used to measure alexithymia. The TAS-20 comprises three subscales, i.e., difficulties identifying feelings (consisting of 7 items), difficulties describing feelings (consisting of 5 items), and externally oriented thinking (consisting of 8 items). The items are rated on a 5-point scale. A TAS-20 total score can be calculated by summing responses to all items.

The Self-Rated Emotional Intelligence Scale (SREIS; German version: Vöhringer et al., 2020) was used to assess emotional intelligence with the facets perception, use, understanding, and regulation of emotion. The SREIS is based on Salovey and Mayer’s (1990) ability-based model of emotional intelligence. The scale consists of five subscales, as the facet emotion regulation is subdivided into the regulation of emotions in other people (Social management) and the regulation of emotions in oneself (Managing emotion (self)). Perceiving emotion comprises four items, referring to the perception and accurate identification of other individuals’ emotions. The Use of emotion scale includes three items related to the ability to harness feelings that assist in cognitive processes such as problem solving, reasoning, and social communication. The Understanding emotion scale comprises four items that refer to the ability to verbally describe and understand feelings. The Managing emotion (self) scale consists of four items related to the ability to modify or control emotional reactions in oneself, and the Social management scale includes four items relating to the ability to regulate other persons’ emotions. Each item is evaluated on a scale ranging from 1 to 5. A total SREIS score can be calculated by summing the subscale scores.

For assessing positive and negative state and trait affects, the Positive and Negative Affect Schedule (PANAS; German version: Krohne et al., 1996) was applied. The PANAS consists of 20 adjectives, 10 describing positive affective states and 10 expressing negative affective states. Participants rated on a 5-point scale how much they feel like this at the moment (state version) or in general (trait version). To assess the level of depressive symptoms experienced over the last two weeks, we administered the Beck Depression Inventory (BDI-II; German version: Hautzinger et al., 2006). The BDI consists of 21 items, with a response format from 0 to 3.

### 2.3. Affective Priming Task: Stimulus Material and Procedure

Face stimuli were monochrome happy, angry, and neutral expressions taken from the Pictures of Facial Affect collection (POFA; Ekman & Friesen, 1976). Neutral and affective faces from ten models (50% women for each face type) were used as primes (see Donges et al., 2015, for examples of prime stimuli). Neutral primes were shown in a vertically mirrored format to avoid identity of the prime and the mask stimulus in the neutral prime condition. A total of 80 experimental trials were presented in the priming task: 20 with happy, 20 with angry, and 20 with neutral prime faces. In 20 trials, primes with no facial features were presented. Each trial was shown twice. In each trial, expressions of the same model were displayed. In the condition of no facial features (“blank” condition), stimuli consisted of neutral faces in which central features, i.e., eyes, nose, and mouth, had been replaced by a surface without contours. Trials were shown in a fixed random sequence, with the constraints that not more than two subsequent trials displayed the same prime category, no two subsequent trials depicted the same model, and no trial was shown twice per half.

Study participants were told to view a series of faces and rate the expression as positive or negative on a 6-point scale ranging from −2.5 to +2.5 by pressing a button on the keyboard. Each trial had a duration of 6 s, in which a prime face was displayed for 33 ms, preceded by a fixation cross shown for 800 ms and followed by a neutral face that was presented for 467 ms. A blank screen followed for 4.700 ms. Two practice trials were shown before the experimental trials. In the practice trials, neutral primes were displayed, and different models were presented than in the experimental trials. Practice trials were not included in the data analysis. Stimulus presentation and response registration were implemented on a Dell Latitude E6500, with a monitor refresh rate of 60 Hz, using Inquisit 3.0 (Draine, 2004).

After the priming experiment, a short interview was conducted in which participants were asked if and what they had noticed during the experiment as well as if and what was displayed before the evaluated faces.

### 2.4. General Procedure

Experimental sessions were carried out individually in a quiet room at the Department of Psychosomatic Medicine and Psychotherapy (University of Leipzig). At the beginning of the testing session, participants’ visual acuity was assessed using a vision chart, followed by the administration of a sociodemographic questionnaire. Questionnaires and priming experiments were given in a fixed sequence: PANAS state, PANAS trait, affective priming experiment, interview on prime awareness, SEC, SREIS, TAS-20, and BDI-II. We opted to administer the behavioral task prior to the self-report questionnaires regarding emotional contagion and competences (i.e., SEC, SREIS, and TAS-20). This was done to prevent priming effects and to ensure that participants’ self-perceptions did not influence their evaluative performance. The study session took approximately 2 h to complete.

### 2.5. Statistical Analysis

A total of 102 study participants had complete data sets. In the case of two participants, data were missing from the BDI-II questionnaire. Mean evaluative ratings were determined for each prime condition. The evaluative data were analyzed in a repeated measures analysis of variance (ANOVA) with one within-subjects factor (prime condition: happy, angry, neutral, and no facial expression). The Greenhouse–Geisser correction (Greenhouse & Geisser, 1959) was applied to correct the degrees of freedom of F-ratios, since the sphericity assumption was violated. To analyze pairwise differences in evaluative ratings between prime conditions, we performed Bonferroni-adjusted pairwise comparisons. Subsequently, affective priming scores were calculated for happy and angry faces by using a single baseline score, i.e., combining the mean evaluative ratings under the neutral and the no-facial-expression prime condition. Affective priming for happy faces was computed by subtracting mean evaluative ratings for neutral mask faces primed by neutral and no facial expression from mean evaluative ratings for neutral mask faces primed by happy faces. In the case of affective priming for angry faces, mean evaluative ratings for neutral mask faces primed by angry faces were subtracted from the mean evaluative ratings for neutral mask faces primed by neutral and no facial expression. These priming scores were used in the subsequent correlation analyses.

Kolmogorov–Smirnov tests were applied to assess normality of distribution. There was a significant departure from normality for the positive and negative SEC subscales (*p*s < 0.01). Against this background, Spearman rank correlation analyses were performed to investigate the relationships of susceptibility to emotional contagion with priming scores, alexithymia, emotional intelligence, affectivity and depressive symptoms. Six correlation analyses were calculated to test our hypotheses concerning the relationships of (positive and negative) susceptibility to emotional contagion with affective priming (based on happy and angry faces, *n* = 2), alexithymia (TAS-20 total score) (*n* = 2), and emotional intelligence (SREIS total score) (*n* = 2). To adjust for multiple testing, we used a corrected *p*-level of 0.0083 (0.05/6). The majority of our correlation analyses were conducted to control confounders and to identify factors associated with susceptibility to emotional contagion. In these exploratory correlation analyses, a *p*-level of 0.05, two-tailed, was administered. Hierarchical regression analyses were calculated if hypothesized relationships were significant, in order to clarify whether these relationships remain significant after adjusting the effects of relevant affect variables, i.e., positive or negative state and trait affect and depressive symptoms. Regression analysis is robust against non-normality, but regression residuals should follow a normal distribution. The multicollinearity of variables was controlled using tolerances and variance inflation factors (Fox, 1991). To test for serial autocorrelation the Durbin–Watson test was applied. To test the robustness of the regression coefficients, bootstrapping with 10,000 resamples was performed, and 95% bias-corrected and accelerated (BCa) confidence intervals were calculated. Statistical analyses were carried out using SPSS software version 31.0 (IBM Corp., Armonk, NY, USA).

Since there are no studies on the relationship between susceptibility to emotional contagion and facial affective priming, well-founded power analyses are difficult to realize. An a priori analysis of statistical power computed with the program G*Power (version 3.1.9.2.; bivariate normal model—exact test family) of Faul et al. (2009) showed that to detect a medium effect of r = 0.3 with an alpha value of 0.05, two-tailed, and a power of 0.80, the required total sample size is 84. Previous findings on the correlation between alexithymia (as assessed by the total scores of the TAS-20 and the BVAQ (Vorst & Bermond, 2001)) and facial affect priming based on angry faces (rs = −0.30 and −0.35) (Rosenberg et al., 2020) indicate that medium effect sizes do not appear unrealistic in this research context, with a personality variable relating to the perception of emotions. Note that alexithymia has been found to be associated with low neural response to facial emotions in various emotion processing areas of the brain (see, e.g., Reker et al., 2010; Van der Velde et al., 2013).

## 3. Results

### 3.1. Affective Priming Task

**Figure 1:**
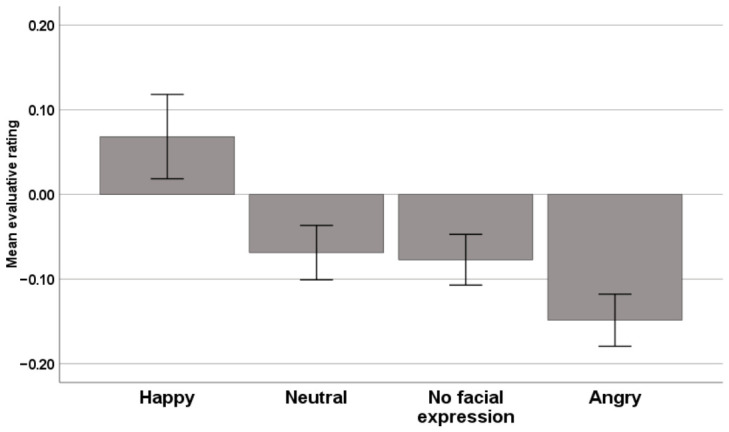
Evaluative ratings as a function of prime condition (happy, neutral, no facial expression, and angry prime) (means with standard errors).

The analysis of the evaluative data included 8.314 responses. In six cases, no data were available because participants did not give an answer within the response window (i.e., 4.700 ms). Mean evaluative ratings were 0.068 (SE = 0.050) for the happy prime condition, −0.069 (SE = 0.032) for the neutral prime condition, −0.077 (SE = 0.030) for the no-facial-expression prime condition, and −0.149 (SE = 0.031) for the angry prime condition (see Figure 1). According to the ANOVA results, there was a significant effect of the prime [F(1.48, 152.92) = 13.74, *p* < 0.0001, partial η^2^ = 0.12]. Bonferroni-adjusted pairwise comparisons indicated that evaluations in the happy prime condition differed significantly from those in the neutral, no-facial-expression, and angry prime condition (*p*s < 0.05). Moreover, evaluations in the angry prime condition differed significantly from those in the neutral and no-facial-expression condition (*p*s < 0.005). Evaluations in the neutral and no-facial-expression condition did not differ from each other.

### 3.2. Prime Awareness

Twenty-eight out of 104 study participants reported to have noticed smiling or laughing faces or faces expressing positive emotions, happiness or joy, that were shown prior to the neutral faces. Two of these participants also reported having seen an angry facial expression prior to the neutral faces. No other participant stated to have seen angry faces. Therefore, it can be assumed that a part of our sample was subjectively aware of the presentation of happy or “positive” faces. When comparing participants with happy prime awareness (*n* = 28) and those without awareness (*n* = 76) on the Positive SEC subscale (4.68 (SD = 0.42) vs. 4.53 (SD = 0.46)), t (102) = 1.53, *p* = 0.13, no significant group differences were revealed.

### 3.3. Relationships of Susceptibility to Emotional Contagion with Affective Priming

The affective priming score for happy faces was 0.141 (SE = 0.042), and the affective priming score for angry faces was 0.076 (SE = 0.017). When comparing participants with happy prime awareness and those without awareness concerning affective priming based on happy faces (0.112 (SE = 0.078) vs. 0.152 (SE = 0.051)), t (102) = −0.41, *p* = 0.68, no significant differences were observed.

The correlation of the Positive SEC scale with the affective priming score based on happy faces was not significant (r = −0.110, *p* = 0.264 (2-tailed), 95% CI [−0.302, 0.090]). The Negative SEC scale was not significantly correlated with the affective priming score based on angry faces (r = −0.013, *p* = 0.899 (2-tailed), 95% CI [−0.210, 0.186]).

We calculated additional correlations in the sample of participants without awareness (*n* = 76). Again, the correlation of the Positive SEC scale with the affective priming score based on happy faces was not significant (r = −0.009, *p* = 0.935 (2-tailed), 95% CI [−0.241, 0.223]). The Negative SEC scale was also not significantly correlated with the affective priming score based on angry faces (r = −0.031, *p* = 0.791 (2-tailed), 95% CI [−0.261, 0.202]).

### 3.4. Relationships of Susceptibility to Emotional Contagion with Alexithymia, Emotional Intelligence, Affectivity, and Depressive Symptoms

As expected, the positive SEC scale was negatively correlated with the TAS-20 total score. At the subscale level of the TAS-20, the positive SEC scale was correlated only with Difficulties describing feelings (see Table 1 for details). However, the negative SEC scale was not correlated with the TAS-20 total score.

**Figure 2:**
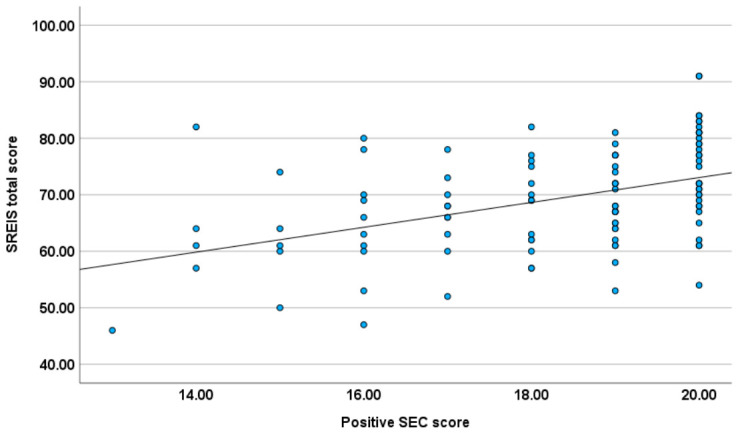
The scatterplot shows the relationship between positive SEC score and SREIS total score (r_s_ = 0.44).

The positive SEC scale was positively correlated with the SREIS total score (see Figure 2). At the subscale level of the SREIS, the positive SEC scale was correlated with all scales, most highly with the scales Perceiving emotion, Understanding emotion, and Social management. The negative SEC scale was not found to be correlated with the SREIS total score. At the subscale level, the negative SEC scale showed only a negative correlation with Managing emotion (self) (see Table 1).

The positive SEC scale was positively correlated with trait positive affect (PANAS) and negatively with level of depressive symptoms (BDI-II), but the positive SEC scale showed no correlations with state and trait negative affect as well as state positive affect. The negative SEC scale was positively correlated with state and trait negative affect (PANAS) and level of depressive symptoms (BDI-II). No correlations were observed between negative SEC and state and trait positive affect (see Table 1).

A regression model for alexithymia (TAS-20 total score) was calculated to examine whether susceptibility to positive emotional contagion is a predictor independent from trait positive affect (PANAS) and depressive symptoms (BDI-II). The first step predicted alexithymia [R^2^ = 0.173, F(2,99) = 10.33, *p* < 0.001], with both trait positive affect and depressive symptoms as significant predictors. In step two, entering positive SEC did not significantly increase the predictive value of the model (see Table 2). In addition, a regression model for emotional intelligence (SREIS total score) was calculated. The initial step predicted emotional intelligence [R^2^ = 0.220, F(2,99) = 13.96, *p* < 0.001], with trait positive affect as a significant predictor. In step two, entering the positive SEC scale significantly increased the predictive value of the model (see Table 3). This means susceptibility to positive emotional contagion was found to be a significant positive predictor of emotional intelligence. The regression residuals of this model were normally distributed (Kolmogorov–Smirnov D (102) = 0.049, *p* > 0.20). The Durbin–Watson test yielded d = 1.94. Tolerance and VIF values indicate no multicollinearity issues in this regression model.

## 4. Discussion

The main objective of our study was to examine whether susceptibility to positive and negative emotional contagion is related to automatic processing of facial emotions. To this aim, we investigated evaluative shifts in response to masked emotional facial expressions using an affective priming task. Affective priming effects reflect a form of susceptibility to being automatically influenced by emotional facial expressions. However, based on our behavioral data, no conclusions can be drawn regarding the mechanisms underlying the observed affective priming effects. We hypothesized that susceptibility to positive emotional contagion is positively correlated with the affective priming effect based on positive primes and susceptibility to negative emotional contagion with the priming effect based on negative primes. This assumption was based on the idea that individuals who are more prone to automatically perceiving and catching others’ emotions may also be more strongly influenced by brief facial expressions of emotions in their judgmental behavior and therefore show greater affective priming. However, our data did not confirm this hypothesis for either positive or negative emotional contagion susceptibility.

Notably, in our task the emotional primes caused valence-congruent shifts in target evaluations across the study group, indicating that primes influenced the perception of the neutral faces. Specifically, neutral targets were evaluated more positively after happy primes and more negatively after angry primes. This confirms that the priming tasks produced the expected effects relative to the baseline condition (neutral faces and faces without facial features). These findings replicate previous research demonstrating affective priming effects with masked facial expressions (e.g., Murphy & Zajonc, 1993; Paul et al., 2012; Sweeny et al., 2009).

Nonetheless, affective priming effects were not related to the personality trait susceptibility to emotional contagion. Although automatic processes of emotion perception are assumed to play an important role in both affective priming and susceptibility to emotional contagion, no correlations between the two constructs were found. One interpretation of this result could be that the neurocognitive mechanisms involved are different. One proposed mechanism underlying emotional contagion is automatic facial mimicry. Spontaneous mimicry of another’s expression generates sensorimotor feedback. This feedback, in turn, triggers a matching internal emotional state (Hatfield et al., 2014; Prochazkova & Kret, 2017; Wróbel & Imbir, 2019). Some evidence suggests that the musculus corrugator supercilii shows affect-congruent responses to masked facial expressions (Rotteveel et al., 2001). However, affective priming effects may not primarily rely on sensorimotor feedback loops or associated feeling responses. Instead, valence-congruent evaluative shifts owing to masked emotional facial expressions could rely on automatic, non-conscious appraisal processes. Semantic information extracted from emotional primes could trigger misattribution processes without requiring an actual feeling response (Rohr et al., 2015; Wentura et al., 2017). Alternatively, masked affective primes may activate emotional representations in memory. These representations then bias the evaluation of subsequent neutral stimuli in a valence-congruent direction (Rohr & Wentura, 2021). Masked facial expressions are processed via a rapid subcortical route involving the amygdala, which can detect emotional significance and is responsive to subtle valence-related cues (Todorov & Engell, 2008; Kim et al., 2017). However, an additional interpretation of the non-correlation results should be taken into consideration. Facial muscle activity was not recorded in our affective priming experiment. This leaves open the question of whether facial mimicry reactions contributed to the emergence of the observed affective priming effects. Based on our behavioral evaluative data, we cannot rule out the possibility that such mimicry was involved in the development of these effects in the present experiment. Should this have been the case, the results would be inconsistent with the assumption that automatic facial mimicry in response to emotional facial expressions contributes to susceptibility to emotional contagion. In summary, our results indicate that individuals with a high susceptibility to emotional contagion do not appear to be particularly sensitive to or influenced by covert facial emotions in their judgment behavior.

When interpreting the observed non-correlation between susceptibility to emotional contagion and affective priming, one should also consider that these two constructs of emotion perception and response differed in their measurement approach. Susceptibility to emotional contagion was assessed via self-report (as is common practice in the research field), reflecting the subjective perception of emotional resonance. In contrast, automatic evaluative shifting was measured using a behavioral performance task. While affective priming seems to be strongly stimulus-driven, the tendency to resonate emotionally with others in everyday life might not only rely on processes of spontaneous facial mimicry but also on the social context and observers’ goals and motivations (Wróbel & Imbir, 2019). Automatic processes of emotional mimicry can be regulated and modified by top-down processes (Hess & Fischer, 2014; Fischer & Hess, 2017). Despite an automatic tendency toward facial mimicry, individuals may engage in corrective efforts to counteract this influence. This often occurs when social norms or personal goals render imitation unsuitable (Wróbel & Imbir, 2019). Against this background, the link between emotional contagion susceptibility and automatic facial response processes might be weaker than originally assumed.

In our study, we applied the recently developed Positive and Negative SEC scales (Marx et al., 2024), which allow for a separate assessment of susceptibility to positive and negative emotional contagion. Marx et al. reported convergent and divergent validity evidence for the SEC scales: they observed, for example, a positive correlation of Positive SEC with positive trait affect (as assessed by the PANAS) and a negative correlation with negative trait affect (as assessed by the PANAS). Furthermore, positive correlations of Negative SEC were found with negative trait affect and measures of depressiveness, but there was no correlation with positive trait affect (Marx et al., 2024). Our study also provides evidence for the construct validity of the SEC scales, as we found a positive correlation between Positive SEC and positive (but not negative) trait affect and a negative correlation of Positive SEC with the level of depressive symptoms (as assessed by the BDI). Moreover, we observed positive correlations of Negative SEC with negative (but not positive) trait affect and depressiveness.

As part of our study, we also investigated the relationships of emotional contagion susceptibility with the personality trait alexithymia. As mentioned previously, alexithymic individuals are characterized by diminished emotional reactivity, showing less physiological arousal and fewer subjective feelings toward emotional stimuli (Vorst & Bermond, 2001; Pollatos et al., 2008). Prior research also highlights their reduced spontaneous facial responses to the emotional expressions of others (e.g., Sonnby-Borgström, 2009; Franz et al., 2021). Our hypothesis that susceptibility to (positive and negative) emotional contagion is negatively correlated with alexithymia was partially confirmed. We found alexithymia (as assessed by the TAS-20 total score) to be negatively correlated with Positive SEC, but not with Negative SEC, indicating that individuals with high susceptibility to positive emotional contagion tend to be less alexithymic. Our results confirm and specify the previous findings of Weisbuch et al. (2011) and Eddy and Hansen (2021). Interestingly, at the subscale level, only difficulties describing (and communicating) feelings correlated negatively with Positive SEC. Other facets, such as difficulties identifying feelings (reflecting interoceptive problems) and externally oriented thinking (a cognitive style focused on external facts rather than emotions), showed no such link to emotional contagion. Our finding is consistent with the observation of Marx et al. (2024) that Positive (but not Negative) SEC is related to interpersonal functioning. The alexithymia facet of difficulties describing feelings has been identified as a significant predictor of interpersonal problems (Spitzer et al., 2005; Koppelberg et al., 2023). However, it must be noted that according to our regression analysis, Positive SEC was not a predictor of alexithymia when affect variables were controlled. This suggests that the correlation between positive emotional contagion and alexithymia can be driven by their common association with mood states rather than a direct psychological mechanism.

Our hypothesis regarding emotional intelligence was partially confirmed. Positive SEC showed a significant positive correlation with the SREIS total score. Thus, the dispositional tendency to catch the positive emotions of others is associated with heightened emotional intelligence. Importantly, according to our regression analysis, susceptibility to positive emotional contagion was a significant predictor of emotional intelligence, independent of positive trait affect and depressiveness. Our findings are in line with those of Clarkson et al. (2025), which showed a positive relationship between emotional intelligence and susceptibility to happiness contagion for women (but not for men). At the subscale level, Positive SEC correlated with all SREIS dimensions. The strongest links emerged for perceiving emotion, understanding emotion, and social emotion management, suggesting that these specific abilities are closely tied to positive emotional resonance. Being more emotionally responsive to positive social signals could lead to better socio-emotional learning and interpersonal understanding. Shared positive emotions are known to improve interpersonal relationship quality, promoting social connectedness, relationship stability, and willingness to cooperate (e.g., Sels et al., 2021; Shiota et al., 2004). However, given the cross-sectional nature of our data, the directionality of the relationship between susceptibility to emotional contagion and emotional intelligence remains unclear. The relationship could be bidirectional: Individuals with high emotional intelligence could be more attuned to social emotional signals and therefore more susceptible to emotional contagion. Conversely, repeated experience and sharing of others’ emotions may enhance emotional competencies through repeated practice in perceiving, interpreting and managing emotional states. Future longitudinal or experimental studies are needed to disentangle these potential causal pathways.

Contrary to expectation, we found no correlation between Negative SEC and emotional intelligence. This result is at least partially not in line with the findings of Clarkson et al. (2025), who reported a negative correlation of contagion susceptibility for the negative emotions fear and anxiety with emotional intelligence. Clarkson et al. (2025) administered the Contagion of Affective Phenomena Scale-Emotion (CAPS-E) to assess emotional contagion, which differentiates between six discrete emotions (i.e., anger, fear, anxiety, sadness, excitement, and happiness). However, in Clarkson et al.’s study, susceptibility to the negative emotions anger and sadness did not correlate with emotional intelligence. Therefore, it is possible that only the tendency to resonate with others’ fear and anxiety states is related to poor emotional competencies. These data imply that fine-grained distinctions in emotional contagion may be relevant to understanding its relations with emotional intelligence.

Our hypothesis that susceptibility to negative emotional contagion is negatively related to emotional intelligence can be questioned for theoretical reasons. High emotional intelligence may not be characterized by a generally low responsivity to or avoidance of others’ negative emotions but rather by the ability to recognize and share the negative emotions of others while maintaining effective regulation strategies. This perspective aligns with the conceptualization of emotional intelligence as involving hypersensitivity to both positive and negative emotions, coupled with regulatory capacity (Fiori et al., 2023; Fiori & Ortony, 2021). According to this model, adaptive emotional functioning depends not on the valence of emotions experienced but on the ability to fully experience emotions and respond appropriately to context (Fiori et al., 2023).

It is important to acknowledge several limitations in our study. First, the generalizability of our findings is limited, as the sample consisted of rather well-educated young women. Given that men and women may differ in emotional processing due to distinct socialization experiences (Brody & Hall, 2008), the results may not be generalizable to men or mixed-gender samples. Previous research has also reported sex differences in susceptibility to emotional contagion (Doherty, 1997; Doherty et al., 1995). Future studies should therefore include both men and women, to examine potential sex differences in susceptibility to emotional contagion. Our findings cannot be generalized to less-educated, older, or clinical populations. Second, in the present study, we found an alpha coefficient of 0.67 for the Positive SEC scale. While this is considered low or modest, it remains acceptable for exploratory purposes. This coefficient is substantially lower than those reported by Marx et al. (2024), who found higher internal consistencies for the scale (i.e., 0.83 in both Study 1 and Study 2). It should be noted that low internal consistency primarily increases the risk of underestimating relationships between variables. The exclusive reliance on self-report measures to assess alexithymia and emotional intelligence is a limitation of our investigation. Self-report measures rely on participants’ subjective self-assessments and may therefore not accurately reflect the constructs they intend to capture, potentially leading to response biases such as social desirability. To address this issue, future studies on the topic could complement self-report measures of alexithymia and emotional intelligence with observer-rated or performance-based assessments to obtain a more comprehensive understanding of the constructs in relation to emotional contagion. While facial mimicry and emotional contagion are related, they are conceptually not identical. Emotional contagion refers primarily to a feeling state, whereas facial mimicry refers to an overt behavior (Hess & Fischer, 2022). Facial mimicry activity seems to contribute only partially to a feeling response (Olszanowski et al., 2020). As discussed above, emotional contagion seems to involve additional mechanisms such as social appraisal (Parkinson, 2011; Wróbel & Imbir, 2019). A further critique of our study could be that a portion of our sample reported to have perceived “smiling” or happy facial primes (even though this awareness of briefly presented positive faces had no influence on affective priming effects) and that awareness of the primes was assessed solely through participant interviews, omitting objective measures such as a prime detection task. However, it should be noted that we did not intend to capture non-conscious or subliminal emotion processing with our priming task. In the present study, we employed only angry and happy faces as prime stimuli. This leaves open the question of whether a relationship may exist between susceptibility to negative emotional contagion and priming effects derived from anxious or sad facial prime stimuli. A further limitation concerns the stimuli: the image collection that we used in our experiment (i.e., the Pictures of Facial Affect set) is dated, and the resolution may no longer meet contemporary viewing standards. Finally, we included no measure of general cognitive ability or processing speed in our study. It cannot be excluded that participants’ general cognitive ability may have introduced systematic variance in how the primes were filtered or integrated, masking to some extent the expected relationship between emotional contagion susceptibility and automatic evaluative processes.

These limitations point to several directions for future research in the field. Future studies might employ test instruments that provide a nuanced assessment of distinct emotion-specific contagion susceptibilities. In this way, one could investigate whether susceptibility to contagion for a specific emotional quality is linked to priming effects driven by that emotion. The differential correlations of susceptibilities to positive and negative emotional contagion with alexithymia and emotional intelligence observed in our study underline the necessity to administer at least bidimensional measures of susceptibility to emotional contagion in future studies. Extreme group comparisons—contrasting individuals with high versus low SEC scores—might help uncover the effects of susceptibility to emotional contagion on affective priming. We recommend that future research investigating the relationship between emotional contagion susceptibility and affective priming incorporates measures of general cognitive ability and processing speed. Although the present study focused on a non-clinical sample, future research might extend this line of inquiry to clinical populations. In a recent study (L. O. Lundqvist, 2026), adults with autism spectrum disorder were found to report lower susceptibility to positive as well as negative emotions than typically developing individuals. A promising research avenue involves investigating the specific relationships between diminished emotional contagion (both positive and negative) and various dimensions of emotional intelligence in individuals with autism spectrum disorder. The role of automatic facial mimicry in emotion contagion and affective priming warrants further investigation. In this context, it would be insightful to administer an affective priming paradigm combined with simultaneous EMG measurement of facial mimicry in order to investigate the relationships of automatic evaluative shifts and automatic mimicry responses with self-reported susceptibility to emotional contagion. We recommend the use of more contemporary face image databases in future priming studies (e.g., the Radboud Faces Database (Langner et al., 2010) or the Karolinska Directed Emotional Faces database (D. Lundqvist et al., 1998)).

## 5. Conclusions

Emotional contagion susceptibility is defined as the tendency to automatically resonate emotionally with others. In our study, we found no evidence that susceptibility to emotional contagion is linked to the automatic processing of and responses to facial emotions as assessed by an affective priming task. It is possible that the neurocognitive mechanisms involved in emotional contagion susceptibility and affective priming differ. However, it can also be argued that the tendency to resonate with other people’s emotions in everyday life might be less strongly related to automatic emotion perception and response than assumed. Instead, emotion contagion susceptibility could be more closely linked to social appraisal processes, which modulate and sometimes inhibit spontaneous facial mimicry according to the observers’ goals and motivations. Furthermore, our research revealed evidence of specific relationships of emotional contagion susceptibility with emotional competences. The tendency to catch others’ positive emotions appears to go along with better abilities in emotion perception, interpretation, and the regulation of others’ emotions, independent of affectivity. Our study’s finding of differential correlations between susceptibility to positive and negative emotional contagion and emotional intelligence indicates that emotional contagion susceptibility should be treated at least as a two-dimensional construct rather than defined and measured as a single dimension.

## Figures and Tables

**Table 1:** Descriptive statistics of and correlations (Spearman rank) between self-report measures with Cronbach’s alpha.

Variable	Positive SEC	Negative SEC	Mean	SD	α
TAS-20 DIF	−0.15	0.33 **	14.71	4.85	0.81
TAS-20 DDF	−0.28 **	0.19	10.64	3.85	0.80
TAS-20 EOT	−0.19	0.01	15.37	4.35	0.74
TAS-20 total score	−0.27 **	0.19	40.73	10.41	0.87
SREIS PE	0.36 **	0.04	15.83	2.21	0.73
SREIS UsE	0.24 *	0.12	10.67	2.37	0.81
SREIS UnE	0.34 **	−0.07	14.01	3.70	0.91
SREIS ME	0.22 *	−0.34 **	13.73	2.43	0.68
SREIS SM	0.35 **	−0.04	14.99	2.65	0.74
SREIS total score	0.44 **	−0.07	69.23	9.16	0.86
PANAS State PA	0.04	−0.01	22.54	4.93	0.78
PANAS State NA	−0.10	0.34 **	3.55	3.73	0.80
PANAS Trait PA	0.36 **	−0.10	26.38	3.99	0.76
PANAS Trait NA	−0.08	0.38 **	6.58	4.79	0.87
BDI-II	−0.25 *	0.23 *	6.49	5.68	0.86

TAS-20, 20-Item Toronto Alexithymia Scale; DIF, difficulties identifying feelings scale; DDF, difficulties describing feelings scale; EOT, externally oriented thinking scale; SREIS, Self-Rated Emotional Intelligence Scale; PE, perceiving emotion scale; UsE, use of emotion scale; UnE, understanding emotion scale; ME, managing emotion (self) scale; SM, social management scale; PANAS, Positive and Negative Affect Schedule; PA, positive affect; NA, negative affect; BDI-II, Beck Depression Inventory. * *p* ≤ 0.05, ** *p* ≤ 0.0083 (two-tailed).

**Table 2:** Hierarchical regression predicting alexithymia (TAS-20 total score) in two steps by positive trait affect (PANAS), depressive symptoms (BDI-II), and positive SEC (*N* = 102).

	Coefficients			Multicollinearity			Model	
Step/Predictor	β	Beta	*t*	Sig. (*p*)	Tol.	VIF	R^2^	∆R^2^
Step 1:								
Positive trait affect	−0.81	−0.32	−3.45	<0.001 ***	0.99	1.01	0.173	-
Depressive symptoms	0.42	0.23	2.53	0.013 *	0.99	1.01		
Step 2:								
Positive trait affect	−0.77	−0.30	−3.04	0.003 **	0.85	1.17	0.174	0.002
Depressive symptoms	0.41	0.23	2.42	0.017 *	0.96	1.04		
Positive SEC	−0.24	−0.04	−0.42	0.673	0.83	1.20		

β = unstandardized regression coefficient; Tol. = Tolerance; VIF = Variance Inflation Factor. * *p* ≤ 0.05, ** *p* ≤ 0.01, *** *p* ≤ 0.001 (two-tailed).

**Table 3:** Hierarchical regression predicting emotional intelligence (SREIS total score) in two steps by positive trait affect (PANAS), depressive symptoms (BDI-II), and positive SEC (*N* = 102) using bootstrapping.

		Coefficients				Multicollinearity			Model	
Step/Predictor	β	SE_b_	Bias	95% BCa CI	Beta	Sig. (*p*)	Tol.	VIF	R^2^	∆R^2^
Step 1:										
Positive trait affect	1.03	0.210	0.004	[0.636, 1.460]	0.45	<0.001 ***	0.99	1.01	0.220	-
Depressive symptoms	−0.15	0.171	−0.013	[−0.509, 0.156]	−0.09	0.306	0.99	1.01		
Step 2:										
Positive trait affect	0.79	0.235	−0.006	[0.336, 1.249]	0.34	<0.001 ***	0.85	1.17	0.288	0.068 **
Depressive symptoms	−0.08	0.159	−0.019	[−0.431, 0.192]	−0.05	0.564	0.96	1.04		
Positive SEC	1.44	0.538	0.008	[0.405, 2.524]	0.28	0.003 **	0.83	1.20		

Results are based on 10,000 bootstrap samples. β = unstandardized regression coefficient; SE_b_ = bootstrap standard error; Bias = bootstrap bias estimate; 95% BCa CI = bias-corrected and accelerated bootstrap confidence interval; Tol. = Tolerance; VIF = Variance Inflation Factor. * *p* ≤ 0.05, ** *p* ≤ 0.01, *** *p* ≤ 0.001 (two-tailed).

## Data Availability

Data available on request due to restrictions (e.g., privacy, legal or ethical reasons).

## Abbreviations

The following abbreviations are used in this manuscript:

BDI-II	Beck Depression Inventory
CAPS-E	Contagion of Affective Phenomena Scale-Emotion
DDF	Difficulties describing feelings
DIF	Difficulties identifying feelings
ECS	Emotion Contagion Scale
EMG	Electromyography
EOT	Externally oriented thinking
ME	Managing emotion (self)
NA	Negative affect
PA	Positive affect
PANAS	Positive and Negative Affect Schedule
PE	Perceiving emotion
POFA	Pictures of Facial Affect collection
SEC	Susceptibility to emotional contagion
SM	Social management
SREIS	Self-Rated Emotional Intelligence Scale
TAS-20	20-Item Toronto Alexithymia Scale
UnE	Understanding emotion
UsE	Use of emotion
